# High Resolution View on the Regulation of Recombinase Accumulation in Mammalian Meiosis

**DOI:** 10.3389/fcell.2021.672191

**Published:** 2021-05-24

**Authors:** Aditya N. Mhaskar, Lieke Koornneef, Alex N. Zelensky, Adriaan B. Houtsmuller, Willy M. Baarends

**Affiliations:** ^1^Department of Developmental Biology, Erasmus MC, Rotterdam, Netherlands; ^2^Oncode Institute, Utrecht, Netherlands; ^3^Department of Molecular Genetics, Erasmus MC, Rotterdam, Netherlands; ^4^Erasmus Optical Imaging Centre, Department of Pathology, Erasmus MC, Rotterdam, Netherlands; ^5^Department of Pathology, Erasmus MC, Rotterdam, Netherlands

**Keywords:** super-resolution microscopy, meiosis, DSB repair, recombinase, ChIP-seq

## Abstract

A distinguishing feature of meiotic DNA double-strand breaks (DSBs), compared to DSBs in somatic cells, is the fact that they are induced in a programmed and specifically orchestrated manner, which includes chromatin remodeling prior to DSB induction. In addition, the meiotic homologous recombination (HR) repair process that follows, is different from HR repair of accidental DSBs in somatic cells. For instance, meiotic HR involves preferred use of the homolog instead of the sister chromatid as a repair template and subsequent formation of crossovers and non-crossovers in a tightly regulated manner. An important outcome of this distinct repair pathway is the pairing of homologous chromosomes. Central to the initial steps in homology recognition during meiotic HR is the cooperation between the strand exchange proteins (recombinases) RAD51 and its meiosis-specific paralog DMC1. Despite our understanding of their enzymatic activity, details on the regulation of their assembly and subsequent molecular organization at meiotic DSBs in mammals have remained largely enigmatic. In this review, we summarize recent mouse data on recombinase regulation *via* meiosis-specific factors. Also, we reflect on bulk “omics” studies of initial meiotic DSB processing, compare these with studies using super-resolution microscopy in single cells, at single DSB sites, and explore the implications of these findings for our understanding of the molecular mechanisms underlying meiotic HR regulation.

## Introduction

Meiotic prophase I in vertebrates begins with programmed induction of DNA double-strand breaks (DSBs) in leptotene [reviewed by Lam and Keeney (2015)]. Somewhat later, coalignment, or pairing, of the homologous chromosomal axes (axial elements) at a distance of ∼400 nm from each other can be observed [reviewed by Zickler and Kleckner (2015)]. As prophase progresses to zygotene, these paired homologs are drawn closer to each other through the assembly of the synaptonemal complex (SC), which physically connects the chromosomal axes at a distance of approximately ∼200 nm (Schücker et al., 2015). The progression of homologous chromosome pairing and synapsis is functionally linked to the concomitant progression of DSBs repair by the specialized meiotic homologous recombination (HR) pathway. By pachytene, the SC assembly (synapsis) along the homologs is complete, and toward the end of pachytene (almost) all meiotic DSBs have been repaired.

Until now, the molecular details of the precise events that lead from meiotic DSB repair to homologous chromosome synapsis have remained obscure. However, advancements in whole-genome analyses and the complementary application of super-resolution microscopy have revealed a plethora of novel information regarding the localization and function of several proteins that are critical to meiotic HR repair and subsequent chromosome pairing in mice (Hinch et al., 2019; Shi et al., 2019; Paiano et al., 2020; Slotman et al., 2020). In this review, we aim to integrate these novel findings to better understand the initial steps of meiotic HR in mice.

## Recombination Initiation During Meiotic Prophase I

In mouse, recombination initiation is triggered by the generation of DSBs by SPO11 and TOPOVIBL in early leptotene (Bergerat et al., 1997; Keeney et al., 1997; Robert et al., 2016a, b). This topoisomerase complex requires several accessory proteins (HORMAD1/REC114/MEI4/MEI1/IHO1/ANKRD31) to ensure (efficient) DSB formation (Kumar et al., 2015, 2018; Stanzione et al., 2016; Boekhout et al., 2019; Papanikos et al., 2019; Acquaviva et al., 2020). According to the model proposed by Kleckner (2006), DSBs occur in chromatin loops after they have become tethered to the chromosomal axis. This axis association of DSB formation is thought to be critical for converting local DNA repair interactions to whole chromosome coalignment during pairing (Zickler and Kleckner, 2015).

Recombination initiation by SPO11/TOPOVIBL is non-random, as was clearly shown by ChIP-seq analyses of meiotic recombinases along with SPO11-oligo sequencing (DNA fragments covalently attached to SPO11 after initial DSB-processing), which have revealed the locations of the many recombination hotspots (Smagulova et al., 2011; Brick et al., 2012). These hotspots are defined by H3K4me3/H3K36me3 signatures, induced by the meiosis-specific histone methyltransferase PRDM9 (Hayashi et al., 2005; Baudat et al., 2010; Berg et al., 2010; Parvanov et al., 2010; Powers et al., 2016). In *Prdm9* knockout mice, DSBs are still induced at other H3K4me3 modified sites, such as enhancers, and the chromatin environment at these aberrant locations may contribute to the impaired DSB repair, causing sterility or reduced fertility depending on genetic background (Hayashi et al., 2005; Brick et al., 2012; Mihola et al., 2019).

## End Processing and Assembly of ssDNA Binding Proteins at the Break Site

Our understanding of break processing following SPO11-mediated recombinase initiation is based on yeast data, in which, Mre11-Rad50-Xrs2 (MRE11-RAD50-NBS1 in *M. musculus* or so-called the MRX/MRN complex) along with Sae2 endonuclease nick the Spo11 bound strand (Mimitou and Symington, 2008; Cannavo and Cejka, 2014). This serves as an entry point for Mre11 mediated 3′–5′ resection and Exo1 and Dna2 mediated 5′–3′ resection, resulting in the release of Spo11 bound oligos and generation of 3′ resected single-stranded DNA (ssDNA) (Neale et al., 2005; Mimitou and Symington, 2008; Zhu et al., 2008; Zakharyevich et al., 2010; Garcia et al., 2011; Keelagher et al., 2011; Cannavo and Cejka, 2014; Mimitou et al., 2017). In mice, DSB processing is thought to be similar with a conserved role for MRN/MRX complex. However, EXO1 appears to be redundant with other long range resection mechanisms (Zhang B. et al., 2020; Paiano et al., 2020; Yamada et al., 2020).

The resulting ssDNA is then bound by the RPA complex and meiosis-specific ssDNA binding proteins SPATA22 and MEIOB (La Salle et al., 2012; Luo et al., 2013; Souquet et al., 2013; Hays et al., 2017). These proteins colocalize extensively when foci first appear in leptotene, their numbers peak in zygotene, and subsequently decline in early pachytene (Souquet et al., 2013; Ishishita et al., 2014; Hays et al., 2017). In fact, MEIOB and SPATA22 form an obligate complex, which facilitates its interaction with subunits of the RPA complex (Xu et al., 2017). However, the functional significance of these interactions is not completely clear, and the recruitment of both RPA and MEIOB/SPATA22 to DSB foci can occur independently (Shi et al., 2019). Interestingly, the absence of MEIOB/SPATA22 has no impact on recombinase recruitment in leptotene (Luo et al., 2013; Ishishita et al., 2014), while in absence of RPA, recombinase loading at meiotic breaks is completely abrogated (Shi et al., 2019). Thus, of these ssDNA binding proteins, only the RPA complex is indispensable for initial recombinase assembly at meiotic DSBs. However, despite normal recombinase recruitment in leptotene, the absence of MEIOB/SPATA22 is associated with a dramatic reduction in RAD51 and DMC1 foci numbers in late zygotene (Souquet et al., 2013; Ishishita et al., 2014). This phenotype is most likely due to a failure to maintain recombinase proteins at the DSBs and not because of faster repair, since the number of RPA foci remains high in *Meiob* and *Spata22* knockout spermatocytes (Luo et al., 2013; Ishishita et al., 2014).

## Regulation of Recombinase Assembly at Meiotic DSBs

As meiosis progresses, ssDNA binding proteins at meiotic DSBs are gradually replaced by the recombinases RAD51 and DMC1 (Moens et al., 2002). Homology search and strand exchange in meiosis are thought to be performed by the meiosis-specific DMC1 while RAD51 plays an accessory role (Cloud et al., 2012; Da Ines et al., 2013; Hong et al., 2013). This idea is mainly based on functional genetic analyses in yeast. Similarly, in mouse, knockout of *Dmc1* leads to a failure to repair meiotic DSBs, causing aberrant and incomplete synapsis in both sexes. Still, RAD51 foci accumulation appears normal (Pittman et al., 1998; Yoshida et al., 1998). Knockout of RAD51 leads to an embryonic lethal phenotype (Lim and Hasty, 1996; Tsuzuki et al., 1996), but an *in vivo* knockdown approach provided indications that DMC1 plays a more dominant role in meiotic DSB repair compared to RAD51 (Dai et al., 2017).

Loading of meiotic recombinase at DSBs sites require several proteins in both somatic and meiotic cells. Here, we restrict ourselves to the components that are most directly involved in the actual transfer of the recombinases onto the ssDNA. Similar to HR in mitotic cells, recombinase loading in meiosis is thought to be directly mediated by BRCA2 (reviewed by Zelensky et al., 2014). However, details of the precise meiotic roles of BRCA2 are missing as knockout of the gene is embryonic lethal (Hakem et al., 1998; Gudmundsdottir and Ashworth, 2004). Nevertheless, *Brca2* knockout mice expressing human BRCA2 rescues embryonic lethality, but the lack of expression in meiotic cells impairs recombinase recruitment and synapsis (Sharan et al., 2004). This confirms the critical role of BRCA2 in recombinase loading in meiosis. In addition, *in vitro* analyses have shown direct interaction between BRCA2 and DMC1 (Dray et al., 2006; Thorslund et al., 2007; Martinez et al., 2016). Thus, it is plausible that the mediator model established in somatic HR is applicable in meiotic HR: BRCA2 delivers RAD51 and DMC1 to meiotic DSBs and facilitates orderly replacement of the ssDNA-binding proteins with the recombinase nucleoprotein filaments. Still, it is unclear how the mediator function of BRCA2 extends to the meiosis-specific ssDNA binding proteins MEIOB/SPATA22 and even to DMC1, as a *Brca2* point mutation affecting the residue essential for BRCA2-DMC1 interaction *in vitro* did not result in the expected meiotic defect (Biswas et al., 2012). Another BRCA2 interactor, SWSAP1, and its partner in the Shu complex SWS1, are both essential for mouse meiosis (Matsuzaki et al., 2019). In absence of either Shu component, RAD51 and DMC1 foci numbers are strongly reduced, which may well be due to reduced stability of the filaments (Abreu et al., 2018; Matsuzaki et al., 2019).

In somatic cells, stable accumulation of RAD51 also requires direct interactions of BRCA2 with its “partner and localizer” PALB2 (Xia et al., 2006). Mouse spermatocytes with a *Brca2* mutation impairing PALB2-BRCA2 interaction displayed a reduced number of RAD51 foci (Hartford et al., 2016). PALB2 forms a complex with the tumor suppressor protein BRCA1, which is thought to help in the accumulation of PALB2 at damage sites (Zhang et al., 2009a, b). Indeed, recombinase accumulation is also impaired in *Brca1* mutant mice (Xu et al., 2003). We and others have recently identified a novel, germ cell specific BRCA2 associated protein complex comprising HSF2BP (or MEILB2) and BRME1 (or MEIOK21/C19orf57), and proposed that this complex functions as meiotic BRCA2 localizer, analogous to the function of PALB2 (Zhang et al., 2019; Brandsma et al., 2019; Felipe-Medina et al., 2020; Shang et al., 2020; Takemoto et al., 2020; Zhang J. et al., 2020). Consistent with this hypothesis, *Hsf2bp* (Brandsma et al., 2019; Zhang et al., 2019) and (to a lesser extent) *Brme1* knockout mice (Zhang J. et al., 2020; Felipe-Medina et al., 2020; Shang et al., 2020) show a strong reduction in meiotic recombinase RAD51/DMC1 foci numbers, associated with severe meiotic defects in males. Interestingly, these proteins are critical only for male fertility while their absence in females has only minor consequences (Zhang et al., 2019; Brandsma et al., 2019; Felipe-Medina et al., 2020; Shang et al., 2020; Takemoto et al., 2020; Zhang J. et al., 2020). This suggests that recombinase loading might be differentially regulated in female mice.

Detection of RPA, SPATA22 and MEIOB in HSF2BP and/or BRME1 co-immunoprecipitates has led to the suggestion that HSF2BP and BRME1 may act as adaptors between BRCA2 and the ssDNA binding protein-coated 3′ overhang of the resected DSB, again not unlike the PALB2 paradigm (Murphy et al., 2014). How PALB2, HSF2BP, and BRME1 divide their BRCA2 localizer roles in meiosis among each other, and how these combine with the intrinsic DNA-binding activity of BRCA2, and its ability to autonomously stimulate recombinases *in vitro* remains to be established. Alternatively, an observed inhibitory effect of HSF2BP on somatic HR caused by its stimulation of proteasomal degradation of BRCA2 (Sato et al., 2020), suggests that HSF2BP (also) affects BRCA2 turnover. The precise functions and regulation of BRCA2 in meiosis may be revealed when better antibodies or other (live) imaging tools for reliable monitoring of BRCA2 abundance and localization in meiocytes become available. An obviously required specialization of BRCA2 in meiosis is the need to assemble RAD51 as well as DMC1 in functional filaments, so it might be speculated that HSF2BP and BRME1 are involved in regulating the meiosis-specific transition from RPA/MEIOB/SPATA22 loaded ssDNA to appropriately assembled RAD51 and DMC1 filaments. But what is the precise molecular organization of these proteins on the ssDNA?

## Molecular Arrangement of RPA, RAD51, and DMC1 Filaments in Nanofoci at Meiotic DSBs

**FIGURE 1:**
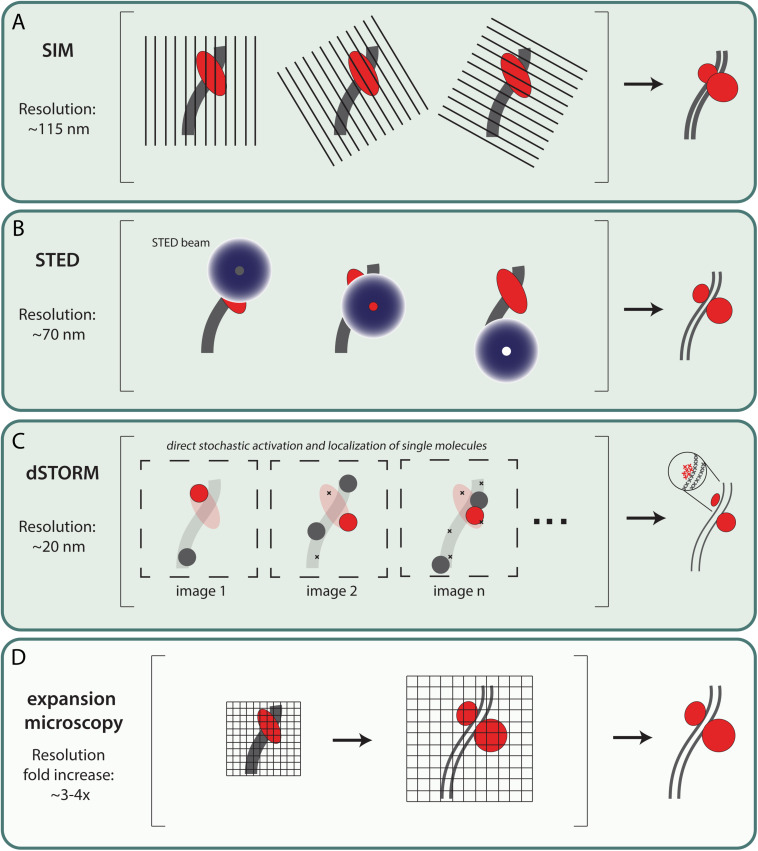
Most commonly used fluorescent super-resolution microscopy techniques in meiosis research. **(A)** Structured illumination microscopy (SIM) – SIM uses a movable diffraction grating in combination with widefield microscopy. By exciting the sample and simultaneously translating and rotating the grid, an interference pattern—also called a Moiré pattern—occurs which contains more detailed information. Using mathematical tools these images are converted to one image with a lateral resolution up to ∼115 nm (Guerra, 1995; Gustafsson, 2000). **(B)** Stimulated emission depletion (STED) – In addition to the excitation laser, STED microscopy includes a second laser that generates a donut shaped STED beam of a longer wavelength that induces stimulated emission of any fluorophore that is not located at the center of the STED beam. This physically narrows the point spread function and therefore increases the lateral resolution up to ∼70 nm (Galbraith and Galbraith, 2011). **(C)** Direct Stochastic optical reconstruction microscopy(dSTORM) – dSTORM microscopy can provide high resolution images with a lateral resolution of ∼20 nm. Fluorophores are stochastically activated by increasing the switch between active and dark-state using an oxygen-reducing buffer and high laser power. Images are generated by combining individual localization signals which are recorded over time (Rust et al., 2006). It should be noted that although single molecules can be detected, the exact number of target proteins present cannot be quantified due to the indirect detection method, using fluorescent antibodies. **(D)** Expansion microscopy (ExM) – Expansion microscopy is a method to increase resolution by enlargement of the sample. This enlargement makes use of a swelling polymer that expand (swelling polymer) samples directly or indirectly (Chen et al., 2015). ExM can be combined with other microscopy techniques resulting in ExM-SIM, ExSTORM or ExSTED with a resolution increase of ∼3–4x fold (Gao et al., 2018; Wang et al., 2018; Xu et al., 2019; Zwettler et al., 2020).

RPA and the recombinases RAD51 and DMC1 are sequentially loaded on the processed ssDNA ends of a DSBs. In this process, the recombinases are thought to replace RPA in the first steps toward strand invasion events. In addition, once strand invasion has been successful, RPA is thought to accumulate on the displaced strand (D-loop). The organization of RPA, RAD51 and DMC1 on meiotic repair intermediates has been analyzed using two different approaches in the mouse. On the one hand, specific ChIP-seq approaches (Smagulova et al., 2011; Hinch et al., 2019, 2020) have been used to study, in bulk cell data, the accumulation of these proteins on ssDNA. On the other hand, there is single cell, and even single repair focus data from fluorescent super-resolution microscopy approaches (Brown et al., 2015; Yoon et al., 2018; Slotman et al., 2020). Fluorescent super-resolution microscopy techniques (for example SIM, STED, dSTORM, and expansion microscopy) bypass the diffraction limit, which restricts the resolution of light microscopy to ∼250 nm (see Figure 1).

In meiosis, initial super-resolution microscopy data was mostly focused on the molecular platform of meiotic DSB repair; the SC (Schücker et al., 2015; Agostinho et al., 2018; Zwettler et al., 2020) and the associated meiotic cohesion components (Agostinho et al., 2016; Ishiguro and Watanabe, 2016; Rong et al., 2016). Structured illumination microscopy (SIM), with a resolution of ∼115 nm (Gustafsson, 2005), is a perfect tool to resolve the lateral elements of the SC, which was not possible with confocal microscopy but earlier shown with electron microscopy (Solari and Moses, 1973; Moses et al., 1975). Using this technique, RPA localization relative to the axial (unsynapsed) and lateral elements of the SC was analyzed by Yoon et al. (2018). They observed that in zygotene, many of the RPA foci localized on the inner side of the still unsynapsed SYCP3 axial elements, and between the synapsed lateral elements in pachytene. These were proposed to represent sites where RPA is associated with D-loops, as also suggested from the ChIP-seq analyses of ssDNA by Hinch et al. (2020). The SIM analyses also allowed a more precise measurement of the foci size which was estimated between 170–270 nm (Yoon et al., 2018). Other super-resolution techniques like STED and dSTORM can obtain even higher resolution, and can visualize relative protein distributions inside the classical repair foci (Hell and Wichmann, 1994; Rust et al., 2006). A further ∼3–4 fold increase in resolution can be achieved by combining expansion microscopy with the above mentioned super-resolution techniques (see Figure 1; Gao et al., 2018; Wang et al., 2018; Xu et al., 2019).

**FIGURE 2:**
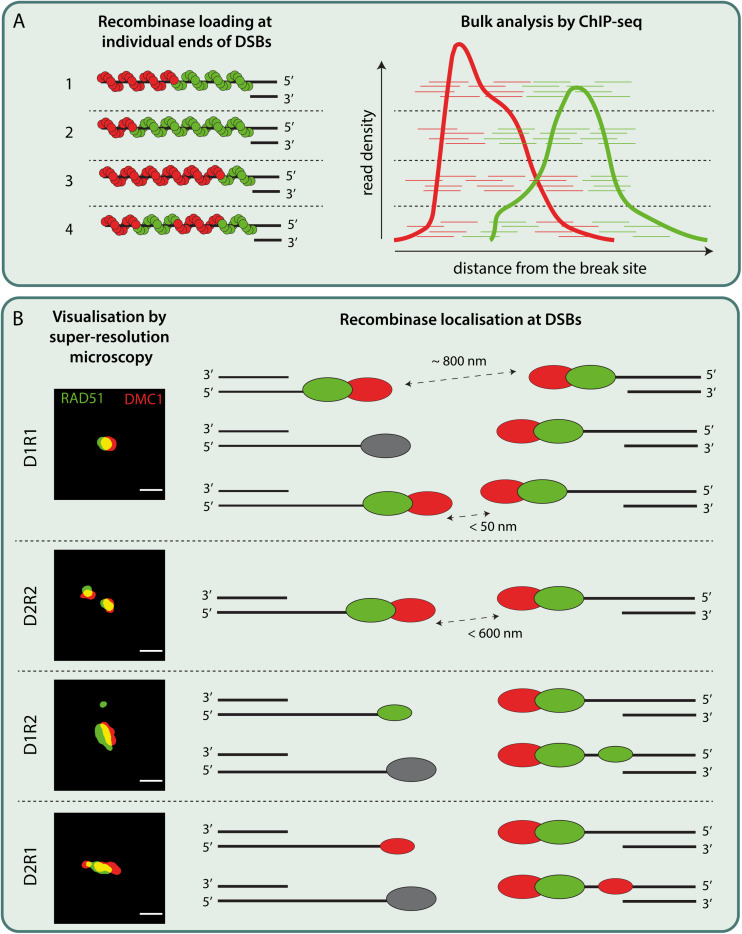
Schematic models interpreting bulk and single-cell data on recombinase loading at individual DSBs in mouse meiosis. **(A)** (left)The location of initial RAD51 and DMC1 loading on ssDNA might be random. The suggested directionality to their filament formation (Brown and Bishop, 2014) could subsequently lead to the RAD51 filaments extending in 3′–5′ direction while DMC1 filaments extending in 5′–3′ direction. The resulting ssDNA would then be occupied with a varying composition of RAD51 and DMC1 recombinases as represented in 1–3. It is equally possible that multiple short stretches of RAD51 and DMC1 filaments are assembled on ssDNA as shown in 4. (right) is a schematic drawing of putative RAD51 and DMC1 ChIP-seq reads corresponding to their loading on individual break sites as depicted in 1–4. The bulk cell nature of ChIP-seq analysis would then yield density plots as indicated in the cartoon, inspired from the RAD51 and DMC1 ChIP-seq analysis from Hinch et al. (2020). **(B)** Super-resolution imaging of DSB repair foci in mice revealed various configurations of DMC1 (D, red oval) and RAD51 (R, green oval). The most frequent configuration, D1R1, can be interpreted in three ways: Assuming that the recombinase foci with a preferred nearest neighbor distance of around ∼800 nm (Slotman et al., 2020) represent two ends of DSB, D1R1 can be interpreted as DMC1-RAD51 pairs loaded symmetrically across two ends of DSB. Alternatively, D1R1 configuration might also be the result of two ends of DSB each loaded with DMC1-RAD51 pairs that end up so close (<50 nm) that they could not be resolved even with super-resolution microscopy. It is equally possible that one end of DSB might be occupied by other ssDNA binding proteins or is being processed while the other end is loaded with DMC1-RAD51 pair. Secondly, based on the paired foci observed in other species and according to ChIP-seq data, a D2R2 can be interpreted as a subset of D1R1 loaded ends of a DSB that lie within the ROI (<600 nm). However, this configuration is rare. Besides these configurations, other configurations such as D2R1 and D1R2 (of which D2R1 is the second most frequent configuration) consisting of a smaller DMC1 or RAD51 foci along with a D1R1 have been observed rather frequently. These configurations may be interpreted as the presence of a single DMC1 or RAD51 focus on the other end of DSB occupied by a D1R1, or an extra focus on the same ssDNA accommodating a D1R1. Other interpretations of these recombinase configurations are also possible. ROI: region of interest Scale bar representing 250 nm. Binary super-resolution images of RAD51 and DMC1 configurations are derived from Slotman et al. (2020).

Localization patterns of RAD51 and DMC1 have been investigated in different species, using different methods. Standard widefield and confocal microscopy imaging have indicated colocalization of RAD51 and DMC1 in mouse meiocytes (Tarsounas et al., 1999; Moens et al., 2002; Carofiglio et al., 2018), but in *A. thaliana*, non-overlapping RAD51/DMC1 foci were observed (Kurzbauer et al., 2012). This has led to the hypothesis that RAD51 and DMC1 were loaded on opposite ends of DSBs. In *C. elegans*, that lacks DMC1, ∼60% of RAD51 was observed in paired foci using 3D-SIM (Woglar and Villeneuve, 2018). Interestingly, in yeast, the appearance of partially overlapping RAD51-DMC1 co-foci has been reported (Brown et al., 2015). Subsequent analyses of RAD51 and DMC1 nanofoci using single color dSTORM, indicated that variable combinations of relatively short (∼100 nm length) RAD51 and DMC1 filaments might be present within a single focus (Brown et al., 2015). However, in mice, high resolution ssDNA ChIP-seq of DMC1 and RAD51 indicated highly organized and symmetric loading of DMC1 near the 3′ ends and RAD51 signal closer to the dsDNA (Hinch et al., 2020). This spatial organization of RAD51 and DMC1 agreed with the SIM data reported by the same group, which showed partially overlapping RAD51-DMC1 foci in close proximity to the SC, where RAD51 was closest (Hinch et al., 2020). Dual color dSTORM analyses in combination with 3D-SIM of mouse spermatocytes also showed that DMC1 is further away from the axis than RAD51 (Slotman et al., 2020). In yeast, this organization was already suggested by the inferred preference of RAD51 to form filaments in 3′–5′ direction, while that of DMC1 would be in 5′–3′ (see Figure 2A, left) from *in vitro* data analysis of directionality in a four-strand reaction (reviewed in Brown and Bishop, 2014). Although the microscopy data at present cannot be translated into an actual organization of filaments on the DNA, combined with the ssDNA ChIP-seq analyses it might be speculated that DMC1 coated ssDNA end has more freedom of movement compared to the RAD51 coated region closer to the dsDNA (and thus to the SC), when searching for homology (Zickler and Kleckner, 2015; Hinch et al., 2020; Slotman et al., 2020). However, contrary to the ChIP-seq data, our super-resolution analysis revealed much less strict-organized RAD51 and DMC1 structures. A bit more than half of the foci in leptotene contain a single DMC1 and a single RAD51 nanofocus (termed D1R1). The second most frequent structure (∼20%) contained two DMC1 nanofoci, and a single, more elongated RAD51 structure (termed D2R1). In addition, the pairing of two RAD51-DMC1 co-foci (two D1R1,D2R1, or other, in any combination) was hardly observed (Slotman et al., 2020). Still, a lack of clear observation of co-foci might be explained if two ends of a DSB are at variable distances. This interpretation would best fit with the expected occurrence of co-foci that each consist of a single DMC1 and RAD51 nanofocus based on the ssDNA ChIP-seq analyses. In addition, or alternatively, (some of the) foci might represent one end of a DSB while the other end is “invisible” because it is still unresected, or associated with other DNA repair factors such as RPA (Paiano et al., 2020). We also cannot exclude that in some foci, the two ends of the DSBs are too close together to be separated even by dSTORM (see Figure 2B). It might be that recombinase loading patterns as described by Hinch et al. (2020) are a consequence of the signal averaging effect that bulk cell analysis could yield (see Figure 2A, combine hypothetical variable recombinase loading patterns shown on the left, with the subsequent expected outcome of ChIP-seq analyses shown on the right). The actual RAD51 and DMC1 loading patterns at individual sites might be more stochastic and thus highly variable (see Figures 2A,B). Nonetheless, both microscopic and omic approaches are consistent with the observation that RAD51 and DMC1 generally form spatially separate structures (Brown et al., 2015; Hinch et al., 2020; Slotman et al., 2020). This implies that BRCA2 somehow loads separate cargos of these proteins on the resected ends. However, the precise mechanism driving this process is still elusive.

Based on *in vitro* analysis and (non-fluorescent) super-resolution imaging in somatic cells, both RAD51 and DMC1 nucleoprotein filament sizes on resected ssDNA could be measured (Lee et al., 2005; Sheridan et al., 2008; Hilario et al., 2009; van Mameren et al., 2009; Candelli et al., 2014; Sanchez et al., 2017; Haas et al., 2018). This data suggests that RAD51 and DMC1 nucleoprotein filaments would occupy ∼100 nt each in yeast meiocytes, which is only a fraction of the estimated 800 nt resected DNA (Brown et al., 2015). Likewise, in mice, simulations based on dSTORM data estimated the average length of RAD51 and DMC1 filaments to be around 140 nm (Slotman et al., 2020). Given the fact that primary and secondary antibodies decorating the underlying protein may add up to 20–40 nm to the resulting image (Mikhaylova et al., 2015; Pleiner et al., 2018), the actual length of each RAD51 and DMC1 filaments would be around 100 nm. Since each nm of recombinase foci corresponds to 2 nt (Ristic et al., 2005; Sheridan et al., 2008; Short et al., 2016), recombinase filaments in mice would occupy around 400 nt of resected DNA. This suggests that only part of 1–2 kb of resected DNA is covered by recombinases (Hinch et al., 2020; Paiano et al., 2020; Yamada et al., 2020). Assuming that the occupation of resected DNA by meiotic recombinase is partial, it can be speculated that RPA, or the other meiosis-specific ssDNA binding proteins (MEIOB/SPATA22) might be simultaneously bound to the rest of ssDNA (Brown et al., 2015; Slotman et al., 2020). However, it cannot be excluded that the actual filament length might be underestimated due to its 3D organization of chromatin in the cell. Measurements related to recombinase occupancy *in vitro* on ssDNA filaments (Sheridan et al., 2008) perhaps cannot be directly “translated” to what is observed *in vivo*, where RAD51 and/or DMC1 loaded DNA might adopt a higher order, or different configuration.

## Concluding Remarks

In this review, we provide an overview of recent data on RAD51 and DMC1 recruitment, which raises several key issues. First, the recently discovered meiotic proteins HSF2BP and BRME1 are critical for BRCA2-mediated loading of RAD51 and DMC1 (Zhang et al., 2019; Zhang J. et al., 2020; Brandsma et al., 2019; Felipe-Medina et al., 2020; Shang et al., 2020; Takemoto et al., 2020). However, key evidence that directly couples BRCA2 localization and activity to that of HSF2BP and BRME1 is still missing. High-resolution imaging of endogenous BRCA2 localization in relation to its proposed recruiters (HSF2BP and BRME1), or genetic experiments that specifically remove interactions between BRCA2 and these proteins are therefore essential.

Second, despite their proposed role in BRCA2 recruitment, the fact that the absence of BRME1 and HSF2BP have only minor consequences in female meiosis is puzzling. This asks for further genetic, biochemical and microscopic analyses of early recombination intermediates in mouse oocytes and comparison with observations in mouse spermatocytes. Third, regarding the differential loading of RAD51 and DMC1, the seemingly strict organization of one DMC1 filament at the 3′ end, and one RAD51 filament further upstream, on both sides of the DSB observed in ChIP-seq data by Hinch et al. (2020), is at odds with the huge diversity of RAD51 and DMC1 nanofoci configurations in super-resolution microscopy (dSTORM) analyses in yeast (Brown et al., 2015) and mice (Slotman et al., 2020). However, the super-resolution microscopy data discussed above is limited by being a 2D snapshot of a dynamic 3D process. Further analysis of recombinases in combination with synaptonemal complex components or even ssDNA using 3D super-resolution techniques could help. Also, it is expected that single cell omics technology involving protein-DNA analyses, will become available, allowing a better comparison of these two types of data. Finally, the development of systems to track a single DSB over time would be another crucial next step to truly unravel the dynamics of the repair process. A recent example of such an approach in somatic cells involved precisely timed and targeted induction of a single DSB, followed by live imaging of repair protein accumulation (Liu et al., 2020). Together, these technical innovations and new approaches will help to make important steps toward our understanding of meiotic recombinase recruitment mechanisms and their functions.

## Author Contributions

AM wrote the draft of the manuscript and designed Figure 2. LK critically reviewed the draft, added text, designed Figure 1, and improved Figure 2. AZ critically reviewed the draft and added text. ABH contributed to the concepts and critically reviewed and adapted the manuscript. WB supervised writing of the draft, and critically reviewed and adapted the manuscript. All authors agreed on the final version of the manuscript.

## Conflict of Interest

The authors declare that the research was conducted in the absence of any commercial or financial relationships that could be construed as a potential conflict of interest.
